# Supplementary material for: Influence of Fetomaternal Microchimerism on Maternal NK Cell Reactivity against the Child’s Leukemic Blasts
Source: Biomedicines. 2022 Mar 4;10(3):603. doi: 10.3390/biomedicines10030603 (PMC8945103; doi:10.3390/biomedicines10030603)
Supplement: Supplementary file 1 [file biomedicines-10-00603-s001.zip › biomedicines-1605662-supplementary.pdf]

Supplementary

Table S1: Correlation of KIR genes and haplotype in child mother and father with the occurrence of FM. Fisher's exact test with risk calculation of odds ratio and 95% confidence analysis was performed. Odds ratios were calculated as follows odds ratio = ((FM-/KIR+)/(FM+/KIR+))/(FM-/KIR-)/(FM+KIR-). Odds ratio >1 = occurrence or absence of gene affects FM. KIR2DL1, KIR2DL4, KIR3DL2 and KIR2DL3 are present in all donors and therefore were excluded. ** Genes are constantly present and therefore were excluded. Significance level p<0.05.

KIR	Child			Mother			Father		
	N	p-value	odds ratio (95% CI)	N	p-value	odds ratio (95% CI)	N	p-value	odds ratio (95% CI)
2DL2	51	0.765	1.388 (0.432-4.459)	51	0.202	0.358 (0.085-1.500)	18	1	0.9 (0.133-6.08)
2DL3	51	0.623	1.765 (0.228-13.685)	51	0.04	6.923 (1.23-35.956)	18	1	0.909 (0.754-1.096)
2DL5	50	0.145	0.368 (0.113-1.198)	51	0.065	0.241 (0.058-0.995)	18	0.627	0.500 (0.068-3.696)
2DP1	**			51	1	0.969 (0.91-1.031)	**		
2DS1	51	0.08	0.306 (0.094-0.998)	51	0.565	0.642 (0.204-2.017)	18	0.627	0.500 (0.068-3.696)
2DS2	51	0.771	0.75 (0.234-2.404)	51	0.543	0.595 (0.171-2.071)	18	1	1.111 (0.164-7.506)
2DS3	44	0.738	1.447 (0.365-5.735)	44	0.456	2.571 (0.475-13.913)	18	1	0.600 (0.031-11.473)
2DS4	51	0.373	1.056 (0.949-1.174)	51	0.373	1.056 (0.949-1.174)	**		
2DS5	51	0.106	0.317 (0.089-1.132)	51	0.563	0.667 (0.211-2.106)	18	1	2.250 (0.185-27.369)
3DL1	51	0.134	1.118 (0.958-1.304)	51	0.373	1.056 (0.949-1.174)	**		
3DP1	0	0.285	0.906 (0.811-1.013)	51	1	0.969 (0.91-1.031)	18	1	0.909 (0.754-1.096)
3DS1	51	0.07	0.311 (0.091-1.065)	51	0.561	0.616 (0.196-1.933)	18	1	1.333 (0.098-18.192)
Haplotype	51	0.128	6 (0.687-52.383)	51	0.236	4.154 (0.4637-37.513)	18	1	1 (0.072-13.868)

Table S2: n values to figures 1 - 6.

Figure	Group		n	
**1 A + B**	female		23	
	male		28	
**1 C + D**	age		51	
**2 A**	**FM$^{+/-}$**	**child**	**mother**	**father**
	FM^{-}	32	32	13
	FM^{+}	19	19	7
**2 B**	**child**	**C1/C1**	**C1/C2**	**C2/C2**
	mother			
	C1/C1	11	4	0
	C1/C2	10	15	7
	C2/C2	0	4	1
**2 C**	**FM$^{+/-}$**	**match**		**mismatch**
	FM^{-} mother	11		21
	FM^{+} mother	14		4
	FM^{-} father	7		6
	FM^{+} father	4		3
**2 D**	match		26	
	mismatch		25	
**2 E + F**	C1/C1 match		11	
	C1/C2 match		15	
	non-self		9	
	missing self		16	
**3 A**	**FM$^{+/-}$**	**2DL3+**		**2DL3-**
	FM^{-}	31		2
	FM^{+}	16		6
**3 B**	all		51	
**3 C**	2DL3 only		8	
	2DL2 and 2DL3		29	
	2DL2 only		14	
**3 D + E**	**FM$^{+/-}$**	**child**		**mother**
	FM^{-}	32		32
	FM^{+}	19		19
**3 F**	**KIR gene motif**		**child**	**mother**
	A/A cen		13	16
	A/B cen		29	31
	B/B cen		9	4

Figure	Group			n	
**3 F**	A/A tel			25	27
	A/B tel			24	23
	B/B tel			2	1
**4**	**KIR**	**FM^{-}**	**FM^{+}**	**FM^{-} act**	**FM^{+} act**
	all act	-	-	42	10
	2DL1/S1	42	10	42	10
	2DL1/S5	30	8	30	8
	2DL1	39	9	39	9
	2DL2/3/S2(DX27)	36	10	36	10
	2DL3	42	10	42	10
	2DL2/3/S2(CH-L)	18	3	18	3
	2DL5	31	9	31	9
	2DS4	41	10	41	10
	2DS3/S4	31	9	31	9
	NKG2A	26	7	26	7
**5 A**	FM^{-}			23	
	FM^{+}			10	
**5 B**	FM^{-}			23	
	FM^{+}			13	
	father			16	
**6 A**		**mother**		**father**	
	match	25		12	
	mismatch	20		9	
**6 B**		**blast**		**K562**	
	HLA-C1/C1	19		15	
	HLA-C1/C2	16		15	
	HLA-C2/C2	4		4	
**6 C**	2DL2+			4	
	2DL2/L3+			19	
	2DL3+			12	
**6 D**	cen A/A			16	
	cen A/B			23	
	cen B/B			5	
	tel A/A			23	
	tel A/B			21	
	tel B/B			0	

Table S3: n-values to supplementary figure 1 -4.

Figure	Group	N		
S1 A	HLA-C	Child	Mother	Father
	C1/C1	23	24	16
	C1/C2	37	38	13
	C2/C2	10	7	4
S1 C	FM+/-	non-self	missing-self	
	FM-	8	13	
	FM+	1	3	
S2	B content score	Child	Mother	Father
	0	15	10	8
	1	27	30	14
	2	26	26	3
	3+4	2	4	3
S3 A	0	7		
	1	21		
	2	15		
	3+4	1		
S3 B	0	0		
	1	4		
	2	7		
	3+4	1		
S3 C	0	4		
	1	13		
	2	6		
	3+4	1		
S3 D	Haplotype	FM-	FM+	
	A	4	0	
	Bx	19	12	

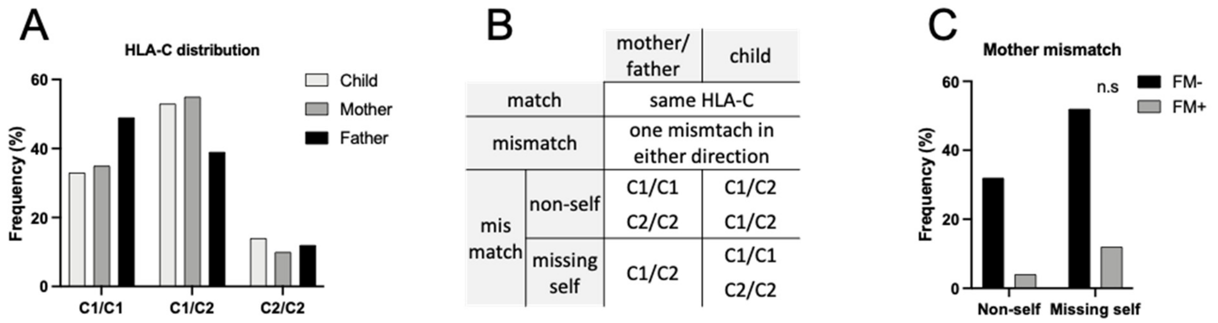

Figure S1: Child, mother and father were HLA-C genotyped and grouped according to their HLA-C genotype into HLA-C1/C1 (C1), HLA-C1/C2 (C1/2) or HLA-C2/C2 (C2). (A) HLA-C distribution of the analyzed cohort. (B) Criteria for grouping depending on HLA match or mismatch of mother and child. (C) Mismatched mothers were grouped into mismatch non-self or missing self. Statistical analysis of frequencies was performed using Fisher's exact test for two groups or Chi2 for more than two. Statistical significance $p<0.05$.

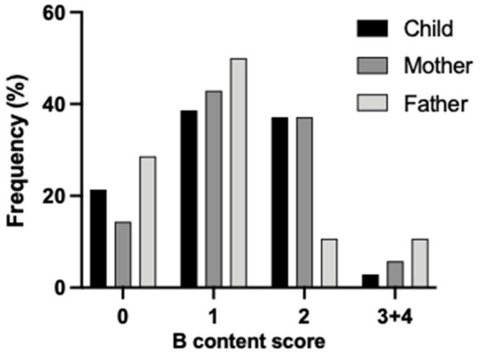

Figure S2: B content score frequencies in the analyzed cohort. B content score was determined after KIR genotyping in child, mother and father. Subsequently, they were grouped depending on their B content score.

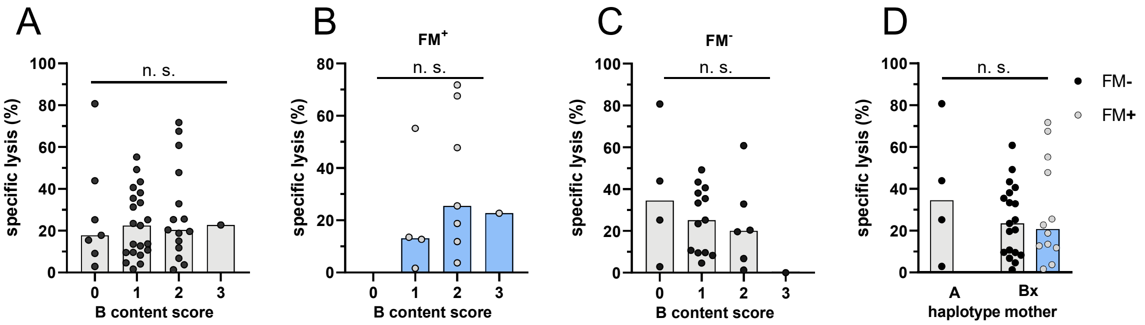

Figure S3: Parental cytotoxicity against filial leukemic blasts. Parental NK cells were isolated and IL-2/IL-15 pre-activated overnight. The next day their cytotoxicity was analyzed against filial blasts and the control cell line K562 in a FACS-based killing assay. (A) All mothers were grouped depending on their B content score and the lysis of leukemic blasts was compared between the groups. Mothers were further grouped into (B) FM+ and (C) FM- and the specific lysis was compared between the groups. (D) Mothers were grouped depending on their KIR haplotype and specific lysis of leukemic blasts was compared. Each dot resembles one sample, bars show mean of the group. Statistical analysis was performed using Kruskal-Wallis test followed by Dunn's multiple comparison test for more than two groups. Significance $p<0.05$, n. s. not significant.
